# Something in the way they move: characteristics of identity present in faces, voices, body movements, and actions

**DOI:** 10.3389/fpsyg.2025.1645218

**Published:** 2025-07-15

**Authors:** Karen Lander, Rachel Bennetts

**Affiliations:** ^1^Division of Psychology, Communication and Human Neuroscience, University of Manchester, Manchester, United Kingdom; ^2^Department of Psychology, College of Health, Medicine, and Life Sciences, Brunel University of London, Uxbridge, United Kingdom

**Keywords:** person identification, biological motion, dynamic characteristics, face, voice, gait, body movements

## Abstract

The recognition of familiar individuals relies not only on static features of the person but also on dynamic characteristics unique to each person’s movements. This mini review synthesizes current research on the role of motion in identity recognition, examining how characteristic dynamic cues from the face, voice, and body may contribute to perceivers’ ability to recognize familiar individuals. We highlight corresponding dynamic covariances that may be present across different aspects of an individual’s motion, such as those linking facial and vocal motion. We evaluate the extent to which dynamic patterns might form a coherent ‘dynamic fingerprint.’ Finally, we consider how variability, distinctiveness, and perceiver-related factors (e.g., individual differences and neural mechanisms) shape the recognition of identity through motion. We outline open questions and propose new directions for understanding the integration of dynamic information in person perception.

## 1 Introduction

The way a person moves reflects their underlying anatomy and changes in the positions of their bones and muscles (Mileva and Burton, 2018; Vick et al., 2007). Yovel and O'Toole (2016) argue that ‘motion acts as the key element for binding together faces, bodies, and voices into a coherent representation of a person that supports recognition’ (p. 383). Indeed, seeing a person move may provide a general ‘form-from-motion’ advantage for recognition, by providing additional views of the person, as well as enhanced structural information about the viewed individual (Johansson, 1973). A complementary idea is that for familiar people, the idiosyncrasies of their observed motion contribute to identity recognition. For example, individuals may have a characteristic smile or way of shaking their head, that serves as a cue to identity. This may also be true of idiosyncrasies present in other aspects of their biological motion, for example gait or gestures (Loula et al., 2005).

## 2 Dynamic characteristics of identity from face and voice

Faces move in rigid and non-rigid ways (Lander et al., 1999). During rigid motion, the face moves as a single object, for example during head nodding and shaking. In contrast, non-rigid motion is deformable or elastic (Xiao et al., 2013), with parts of the face moving in relation to one another, for example when expressing, talking etc. Individuals vary in the amount and way they move their faces, and this can influence how they are perceived by others. Indeed, more facially expressive participants are rated as being more likeable, agreeable, and successful (Cavanagh et al., 2024). Conversely, people who do not move their faces very much are often perceived as uninterested. For example, see work on the impact of reduced facial expression in Parkinson’s (Tickle-Degnen et al., 2011).

Research has shown that seeing a face move leads to better face matching (speaking, Bennetts et al., 2013; expressions, Thornton and Kourtzi, 2002), learning of unfamiliar faces (speaking, Butcher et al., 2011; non-rigid and rigid, Lander and Bruce, 2003; rigid, Pike et al., 1997) and identification of familiar faces (speaking, Butcher and Lander, 2017; non-rigid, Lander et al., 2001). The ‘movement advantage’ may be particularly pronounced when viewing conditions are difficult (Lander et al., 2001) or there is reduced recognition by the observer due to impairment (Bennetts et al., 2015; Longmore and Tree, 2013) or age (Otsuka et al., 2009; Xiao et al., 2014). Dynamic cues may be used flexibly when static cues are insufficient for identification, with facial form and motion information optimally integrated to support recognition (Dobs et al., 2017).

Seminal work by O'Toole et al. (2002) formalizes, for faces, the distinction between a general advantage of motion and one linked to the characteristics of the observed motion. Indeed, the ‘representation enhancement hypothesis’ (O'Toole et al., 2002; O'Toole and Roark, 2011) suggests that seeing a face move aids recognition by facilitating perception of the three-dimensional face structure. Here, there is thought to be a generic benefit (over any advantage of multiple statics) of seeing a face move, that is useful both when learning a face or when recognizing it (Pike et al., 1997; Butcher et al., 2011). The ‘supplemental information hypothesis’ (O'Toole et al., 2002) proposes that we represent characteristic facial motions of individual faces, in addition to the invariant structure of the face. These characteristic facial motions are referred to as ‘dynamic identity signatures’ (Simhi and Yovel, 2020) and are typically found through characteristic expressions, manner of speaking (Dobs et al., 2016) or ways of looking (Peterson et al., 2025). This theory is supported by studies that manipulate the temporal characteristics of the observed facial motion by slowing, speeding or reversing clips (e.g., Lander and Bruce, 2000; Lander et al., 2006). These manipulations disrupt the characteristic patterns of movement and reduce the movement advantage for familiar faces. Such characteristic information may be inherent to ‘dynamic’ representations (Freyd, 1987) or be stored alongside a static-based representation.

Importantly, dynamic facial signatures are thought to be learnt over time, providing a reliable cue to identity for familiar faces, and one that is increasingly useful the more familiar the face is. Accordingly, Butcher and Lander (2017) found that the magnitude of the motion advantage observed for an individual face correlated with how familiar that face was (but see Bennetts et al., 2013). Further, more distinctive facial movement patterns were associated with a greater movement advantage in familiar faces (Lander and Chuang, 2005). Here, distinctive refers to movement characteristics that differ from average or typical movements – they are unique, unusual, or idiosyncratic to an individual. This finding supports the idea that dynamic facial signatures are more relevant for familiar than unfamiliar face recognition.

Interestingly, dynamic characteristics in the way a face moves may also be present in the way a person sounds (Kamachi et al., 2003; Lander et al., 2007; Munhall and Buchan, 2004). Kamachi et al. (2003) found that participants could match unfamiliar faces to voices (or voices to faces) above chance and that matching performance was best with dynamic face stimuli (but see Lavan et al., 2021, who found chance-level dynamic face-voice matching). Face to voice matching tasks demonstrate that dynamic covariances of identity are present in the movement of faces and voices. Similar to visual-only dynamic identity signatures, these identity covariances are likely based on relative timing information: reversing or transforming speech in a non-linear manner disrupts cross-modal matching performance (Lachs and Pisoni, 2004a,b).

## 3 Dynamic characteristics of identity from body movement and actions

Body motion is a pivotal factor in human perception and the recognition of identity (Troje, 2002). Perceivers use body motion to help categorize others’ social identities, and these categorizations may carry important consequences such as mate selection (Lick et al., 2013) and prejudice (Johnson et al., 2007). Simplistically, non-rigid body motion can be categorized into: (i) biological motion, which refers to the natural movements of people, like gait or gestures and (ii) motions associated with specific purposeful activities like drinking or sports type actions (Dittrich, 1993).

One of the most studied aspects of body motion in identity recognition is gait analysis. Gait refers to an individual’s unique pattern of walking (Whittle, 2007), which possesses measurable properties that remain consistent over time, are observable from a distance and difficult to camouflage (Zhang et al., 2011). These individualized parameters include stride length, step frequency, limb movement, posture and rhythm, which may be used as biometric markers for identity verification or identification, especially within automated security settings (Bastos and Tavares, 2025).

Early work focusing on the recognition of identity from gait used point-light displays (PLDs), where ‘lights’ are placed on key areas of the body with all other visual cues removed. When static the image appears like a collection of spots, but when the image moves the body becomes apparent. Cutting and Kozlowski (1977) showed that participants were able to correctly identify an individual walker from six friends 38% of the time (also see Troje et al., 2005). Loula et al. (2005) asked participants to make forced choice decisions about whether a PLD was displaying themselves, a friend or a stranger. Results found that self-recognition was best (69% correct) with friend recognition also significantly above chance. Interestingly, the greatest advantage for accurate recognition of self was from more expressive movements like dancing and boxing.

Further work on the role of gait in identity recognition has used impoverished ‘natural’ image sequences. For example, Stevenage et al. (1999) found that participants were able to use gait to distinguish between six individuals. Additionally, Baragchizadeh et al. (2020) found that participants’ were able to make identity matching decisions to unfamiliar people performing the same action (e.g., both walking) or different actions (e.g., walking and boxing) above chance. Further, Simhi and Yovel (2017) asked participants to study people in motion and recognise them from dynamic or multi-static images. Results suggested that dynamic identity signatures may contribute to person recognition, but only of familiar people previously seen in motion. Finally, Simhi and Yovel (2020) used a virtual reality recognition memory task, with participants learning dynamic identities at study. At test, images were shown dynamic or as a series of multi-statics. At test, dynamic identities with distinctive gaits were recognized more accurately, from a greater distance away, compared to less distinctive walkers. No such effect was found in the multi-static condition, highlighting the importance of dynamic gait to person recognition.

Beyond gait, other elements of body motion, such as hand gestures, posture shifts, and head movements, may also contribute to identity recognition (Pilz and Thornton, 2016). Hand gestures facilitate communication for both the speaker and listener (Wagner et al., 2014). They are known to be idiosyncratic, influenced by cultural and personal habits (Gawne, 2025), making them distinguishable between individuals (see Gillespie et al., 2014). Further, exaggeration of body actions may be particularly important for identification of an individual (Hill and Pollick, 2000). It seems likely that when viewing bodies in motion we are able to use characteristic motion signatures to help identify the individual shown. To summarize, research has established a beneficial role of motion when recognizing familiar people from body movements and actions, centered around idiosyncratic patterns of movement that aid identification.

## 4 Considerations and future directions

We have outlined the sources of evidence that support the idea of characteristic motion patterns, useful in the recognition of identity. Such characteristics seem to present in the movement of our faces, voices, bodies and actions. Several issues for consideration remain.

First, we need to understand more clearly what we exactly mean by ‘characteristic motion patterns’. Here, it is not clear whether ‘characteristic’ is synonymous with ‘distinctive’ – in other words, whether characteristic movement patterns need to be unusual or unique to the individual in some way to support recognition. One way to better understand the extent to which the two parameters are related is to examine how variation in the distinctiveness of movement patterns affects the movement advantage. Some people naturally move more distinctively than others, which may mediate the size of any motion advantage (Lander and Chuang, 2005), supporting a possible effect of natural between-person variability in distinctiveness. Other studies have examined whether the movement advantage is affected by manipulating distinctiveness artificially. As with spatially-based distinctiveness (Valentine, 1988), we can also manipulate the distinctiveness of observed motion by caricaturing motion relative to a ‘norm’. Furl et al. (2022) used a face space account (Valentine, 1991) where the axes in the multi-dimensional space reflect spatiotemporal dimensions such as speed, displacement, and relative timing. In this work, spatiotemporal caricatures of unfamiliar faces had a minimal effect on identity processing, regardless of whether presented at learning or test. In contrast, Hill and Pollick (2000) found a benefit of caricatures for the recognition of body motion. They trained participants to recognise individuals’ arm movements, and then tested them on temporally exaggerated movements made by the same actors. Recognition levels were higher for increasing levels of exaggeration, suggesting that time-based cues were important for identification. Further studies with familiar people and matched methodologies are required to compare the role of distinctiveness of movement cues in face and body identification. Current disparate findings raise the possibility that movement cues might be integrated into identity judgements differently for faces and bodies – at least, when they are unfamiliar.

Second, we should also consider whether there are common dynamic characteristics found across different aspects of a person that are identity specific – a dynamic fingerprint, if you like, that acts as a cue to identity. Research has generally supported a link between visible face motion and the audible sound of the voice, although it is important to note that some people look and sound more similar than others (Smith et al., 2016). But what about other possible links between person specific motion? At the most basic level, for example, does a person who has particularly pronounced facial movements also have similar style body movements. Future work needs to look at whether such commonalities in motion exist – and if they do, what they look like – and how they might be used to create a dynamic fingerprint that aids identification of a person. Future work may explore between-person variability in the usefulness of dynamic signatures for identification. As reviewed above, there is preliminary evidence that some between-person variability in movement characteristics affects the extent to which they benefit recognition (Lander and Chuang, 2005), but there is little research on other factors that might make some people easier to recognise than others (or, conversely, that lead us to perceive their motion as very similar). Here, multivariate time-series modelling of the dynamic parameters of the whole person may facilitate intra- and inter-subject comparison of dynamic movement patterns (Joo et al., 2018). Understanding the relative reliability and usefulness of dynamic information from the face, body, and voice when making identity judgements – and how this relates to their actual use in identification scenarios – could inform human- and computer-based person identification. Crucially, research investigating the integration of different cues (e.g., face and body) needs to focus specifically on moving stimuli: previous work has shown that people allocate attention to faces and bodies differently when they are static (attention primarily to the face) and dynamic (attention to both the face and body) (O’Toole et al., 2011).

Further, in order for dynamic fingerprints to be useful for recognition, we might expect these to be relatively stable across time and context. However, as well as being more useful for some compared with others, the movement of a person might also vary between different viewing instances of the same person. On some occasions a person might move in their typical way, whereas on other occasions they may not. For example, they may be tired, flattening the characteristics of their observed motion. Alternatively, people might naturally exaggerate their movements, either intentionally (e.g., overenunciating speech) or unintentionally (e.g., intense emotional expressions). Surprisingly little work has addressed how this natural variation affects characteristic motion patterns: for example, whether it increases or hinders the usefulness of movement cues for identification, or whether there are certain dynamic cues that remain consistently available across situations. In the domain of emotion recognition, the increased physical movements associated with higher emotional intensity improve emotion recognition performance (e.g., Hess et al., 1997), but to date there has been no research directly examining the effects of natural variations (exaggerations or reductions) of movement on identification.

Third, if we accept the idea of dynamic fingerprints then we need to consider where such information is integrated in the brain. Early neural models of person perception drew a distinction between the processing of invariant and changeable aspects of a person (Haxby et al., 2000). Invariant features like identity were thought to be processed in the occipital and fusiform face areas (OFA and FFA) and the fusiform and extrastriate body areas (FBA and EBA). Whereas the processing of changeable aspects of a person (like eye gaze, expressions etc.) were linked to the posterior superior temporal sulcus (pSTS; O'Toole et al., 2002; Yovel and O'Toole, 2016). Importantly, research has shown the pSTS responds more strongly to dynamic than static faces, while the FFA and OFA show similar responses to static and dynamic faces (Pitcher et al., 2011; Bernstein et al., 2018). The pSTS is also strongly activated in response to biological motion and to human voices and audiovisual speech (Deen et al., 2015), making it likely that this area plays a key role in the processing and integration of dynamic fingerprints (Yovel and O'Toole, 2016). However, the pSTS is likely only one part of a broader network involved in dynamic person representations. Preliminary evidence (with emotional face expressions) suggests that spatiotemporal facial cues may be represented throughout the face-selective and motion-selective networks in the brain in a spatiotemporal version of ‘face space’ (Furl et al., 2020). Other work has found a relationship between biological motion perception and activation in both pSTS and the ventral premotor cortex (Gilaie-Dotan et al., 2013). Further work examining other forms of facial, biological, and cross-modal dynamic information (Küçük et al., 2024) is needed to confirm the regions, interactions, and mechanisms involved in processing whole-person dynamic cues.

Finally, research needs to take individual differences of perceivers into account when considering the usefulness of dynamic fingerprints for identification. It is well-established that some people are better at static face identification than others (Wilmer, 2017); likewise, there is individual variation in biological motion perception (Miller and Saygin, 2013), and the movement advantage for face recognition (Butcher and Lander, 2017). However, the extent and consistency of individual differences in the movement advantage have not yet been examined. Interestingly, individuals with prosopagnosia – a severe deficit in face recognition – still show a movement advantage for faces (Bennetts et al., 2015; Longmore and Tree, 2013; Steede et al., 2007). This supports the idea, discussed above, that movement cues might act as a complementary source of information when static cues are less reliable. Super-recognizers, who show exceptional face recognition ability (Russell et al., 2009), also show a movement advantage for famous face recognition (Davis et al., 2016). Thus, findings suggest that the ability to extract and use static cues to identity does not align directly with the ability to extract and use facial movement as a cue to identity (notably, there is also no relationship between static face recognition and identification of biological motion in bodies; Noyes et al., 2018). Nor can the movement advantage be linked to underpinning visual processing strategies: recent work found no association between the movement advantage for famous face recognition and differences in eye-movements to static and dynamic faces (Butcher et al., 2025). It may be that other factors, such as sensitivity to biological motion or other spatiotemporal information, might predict individual differences in this skill. Research into these factors, applying not only to faces but recognition of identity from other aspects like gait, body movement etc. is needed. The development of reliable and consistent measures of individual differences in identifying dynamic signatures may be particularly important in applied contexts, where it may be useful to screen for individuals who excel at specific recognition-based tasks (e.g., identifying known suspects on poor-quality video footage; Bate et al., 2021).

## 5 Conclusion

This mini review explores how characteristic motions contribute to recognizing familiar people. Movement provides structural and identity-specific cues that enhance recognition, especially under challenging conditions or when static information is limited. Research shows that individuals have dynamic identity signatures, which are learned over time and aid recognition. These cues may be consistent across face, body, and voice, forming a ‘dynamic fingerprint.’ However, more research is needed to clarify the importance of distinctiveness, how stable these motion cues are between- and within-people, how they are processed in the brain, and how individual differences in perceiver affect their use.

## References

[ref1] Baragchizadeh A. Jesudasen P. R. O'Toole A. J. (2020). Identification of unfamiliar people from point-light biological motion: a perceptual reevaluation. Vis. Cogn. 28, 513–522. doi: 10.1080/13506285.2020.1834039

[ref2] Bastos D. R. M. Tavares J. M. R. S. (2025). Advances in gait-based identification: A systematic review of deep learning models leveraging computer vision techniques. Switzerland: Springer. Available at online: https://link.springer.com/book/10.1007/978-3-031-89560-9

[ref3] Bate S. Portch E. Mestry N. (2021). When two fields collide: identifying “super-recognisers” for neuropsychological and forensic face recognition research. Q. J. Exp. Psychol. 74, 2154–2164. doi: 10.1177/17470218211027695, PMID: 34110226 PMC8531948

[ref4] Bennetts R. J. Butcher N. Lander K. Udale R. Bate S. (2015). Movement cues aid face recognition in developmental prosopagnosia. Neuropsychology 29, 855–860. doi: 10.1037/neu0000187, PMID: 25822463

[ref5] Bennetts R. J. Kim J. Burke D. Brooks K. R. Lucey S. Saragih J.. (2013). The movement advantage in famous and unfamiliar faces: a comparison of point-light displays and shape-normalised avatar stimuli. Perception 42, 950–970. doi: 10.1068/p7446, PMID: 24386715

[ref6] Bernstein M. Erez Y. Blank I. Yovel G. (2018). An integrated neural framework for dynamic and static face processing. Sci. Rep. 8:7036. doi: 10.1038/s41598-018-25405-9, PMID: 29728577 PMC5935689

[ref7] Butcher N. Bennetts R. J. Sexton L. Barbanta A. Lander K. (2025). Eye movement differences when recognising and learning moving and static faces. Q. J. Exp. Psychol. 78, 744–765. doi: 10.1177/17470218241252145, PMID: 38644390 PMC11905330

[ref8] Butcher N. Lander K. (2017). Exploring the motion advantage: evaluating the contribution of familiarity and differences in facial motion. Q. J. Exp. Psychol. 70, 919–929. doi: 10.1080/17470218.2016.1138974, PMID: 26822035

[ref9] Butcher N. Lander K. Fang H. Costen N. (2011). The effect of motion at encoding and retrieval for same- and other-race face recognition. Br. J. Psychol. 102, 931–942. doi: 10.1111/j.2044-8295.2011.02060.x, PMID: 21988393

[ref10] Cavanagh K. Whitehouse H. Waller B. M. (2024). Being facially expressive is socially advantageous. Sci. Rep. 14:62902. doi: 10.1038/s41598-024-62902-6 PMC11176176 38871925

[ref11] Cutting J. E. Kozlowski L. T. (1977). Recognizing friends by their walk: gait perception without familiarity cues. Bull. Psychon. Soc. 9, 353–356. doi: 10.3758/BF03337021

[ref12] Davis J. P. Lander K. Evans R. Jansari A. (2016). Use-inspired basic research on individual differences in face identification: implications for criminal investigation and security. Front. Psychol. 7:219. doi: 10.3389/fpsyg.2016.00219 29984301 PMC6021459

[ref001] Deen B. Koldewyn K. Kanwisher N. Saxe R. (2015). Functional Organization of Social Perception and Cognition in the Superior Temporal Sulcus, Cerebral Cortex 25, 4596–4609. doi: 10.1093/cercor/bhv111 26048954 PMC4816802

[ref13] Dittrich W. H. (1993). Action categories and the perception of biological motion. Perception 22, 15–22. doi: 10.1068/p220015, PMID: 8474831

[ref14] Dobs K. Bülthoff I. Schultz J. (2016). Identity information content depends on the type of facial movement. Sci. Rep. 6. doi: 10.1038/srep34301, PMID: 27683087 PMC5041143

[ref15] Dobs K. Ma W. J. Reddy L. (2017). Near-optimal integration of facial form and motion. Sci. Rep. 7:11002. doi: 10.1038/s41598-017-10885-y, PMID: 28887554 PMC5591281

[ref16] Freyd J. J. (1987). Dynamic mental representations. Psychol. Rev. 94, 427–438. doi: 10.1037/0033-295X.94.4.427 3317470

[ref17] Furl N. Begum F. Ferrarese F. P. Jans S. Woolley C. Sulik J. (2022). Caricatured facial movements enhance perception of emotional facial expressions. Perception 51, 313–343. doi: 10.1177/0301006622108645 35341407 PMC9017061

[ref18] Furl N. Begum F. Sulik J. Ferrarese F. P. Jans S. Woolley C. (2020). Face space representations of movement. NeuroImage 212:116676. doi: 10.1016/j.neuroimage.2020.116676, PMID: 32112962

[ref19] Gawne L. (2025). “Gesture across cultures and languages” in Gesture: A Slim Guide (Oxford, UK: Oxford Academic).

[ref20] Gilaie-Dotan S. Kanai R. Bahrami B. Rees G. Saygin A. P. (2013). Neuroanatomical correlates of biological motion detection. Neuropsychologia 51, 457–463. doi: 10.1016/j.neuropsychologia.2012.11.027, PMID: 23211992 PMC3611598

[ref21] Gillespie M. James A. N. Federmeier K. D. Watson D. G. (2014). Verbal working memory predicts co-speech gesture: evidence from individual differences. Cognition 132, 174–180. doi: 10.1016/j.cognition.2014.03.012, PMID: 24813571 PMC4066192

[ref22] Haxby J. V. Hoffman E. A. Gobbini M. I. (2000). The distributed human neural system for face perception. Trends Cogn. Sci. 4, 223–233. doi: 10.1016/s1364-6613(00)01482-0, PMID: 10827445

[ref23] Hess U. Blairy S. Kleck R. E. (1997). The intensity of emotional facial expressions and decoding accuracy. J. Nonverb. Behav. 21, 241–257. doi: 10.1023/A:1024952730333

[ref24] Hill H. Pollick F. E. (2000). Exaggerating temporal differences enhances recognition of individuals from point light displays. Psychol. Sci. 11, 223–228. doi: 10.1111/1467-9280.00245, PMID: 11273407

[ref25] Johansson G. (1973). Visual perception of biological motion and a model for its analysis. Percept. Psychophys. 14, 201–211. doi: 10.3758/BF03212378

[ref26] Johnson K. L. Gill S. Reichman V. Tassinary L. G. (2007). Swagger, sway, and sexuality: judging sexual orientation from body motion and morphology. J. Pers. Soc. Psychol. 93, 321–334. doi: 10.1037/0022-3514.93.3.321, PMID: 17723051

[ref27] Joo H. Simon T. Sheikh Y.. (2018). Total capture: a 3D deformation model for tracking faces, hands, and bodies. Proceedings of the IEEE/CVF Conference on Computer Vision and Pattern Recognition (CVPR 2018), 8320–8329

[ref28] Kamachi M. Hill H. Lander K. Vatikiotis-Bateson E. (2003). 'Putting the face to the voice': matching identity across modality. Curr. Biol. 13, 1709–1714. doi: 10.1016/j.cub.2003.09.005 14521837

[ref29] Küçük E. Foxwell M. Kaiser D. Pitcher D. (2024). Moving and static faces, bodies, objects, and scenes are differentially represented across the three visual pathways. J. Cogn. Neurosci. 36, 2639–2651. doi: 10.1162/jocn_a_02139, PMID: 38527070 PMC11602004

[ref30] Lachs L. Pisoni D. B. (2004a). Cross-modal source information and spoken word recognition. J. Exp. Psychol. Hum. Percept. Perform. 30, 378–396. doi: 10.1037/0096-1523.30.2.378, PMID: 15053696 PMC3432944

[ref31] Lachs L. Pisoni D. B. (2004b). Crossmodal source identification in speech perception. Ecol. Psychol. 16, 159–187. doi: 10.1207/s15326969eco1603_1, PMID: 21544262 PMC3085281

[ref32] Lander K. Bruce V. (2000). Recognizing famous faces: exploring the benefits of facial motion. Ecol. Psychol. 12, 259–272. doi: 10.1207/S15326969ECO1204_01

[ref33] Lander K. Bruce V. (2003). The role of motion in learning new faces. Vis. Cogn. 10, 897–912. doi: 10.1080/13506280344000149

[ref34] Lander K. Bruce V. Hill H. (2001). Evaluating the effectiveness of pixelation and blurring on masking the identity of familiar faces. Appl. Cogn. Psychol. 15, 101–116. doi: 10.1002/1099-0720(200101/02)15:1<101::AID-ACP697>3.0.CO;2-7

[ref35] Lander K. Christie F. Bruce V. (1999). The role of movement in the recognition of famous faces. Mem. Cogn. 27, 974–985. doi: 10.3758/BF03201228 10586574

[ref36] Lander K. Chuang L. (2005). Why are moving faces easier to recognize? Vis. Cogn. 12, 429–442. doi: 10.1080/13506280444000382

[ref37] Lander K. Chuang L. Wickham L. (2006). Recognizing face identity from natural and morphed smiles. Q. J. Exp. Psychol. 59, 801–808. doi: 10.1080/17470210600576136, PMID: 16608747

[ref38] Lander K. Hill H. Kamachi M. Vatikiotis-Bateson E. (2007). It's not what you say but the way you say it: matching faces and voices. J. Exp. Psychol. Hum. Percept. Perform. 33, 905–914. doi: 10.1037/0096-1523.33.4.905, PMID: 17683236

[ref39] Lavan N. Smith H. Jiang L. McGettigan C. (2021). Explaining face-voice matching decisions: the contribution of mouth movements, stimulus effects and response biases. Atten. Percept. Psychophys. 83, 2205–2216. doi: 10.3758/s13414-021-02290-5, PMID: 33797024 PMC8213568

[ref40] Lick D. J. Johnson K. L. Gill S. V. (2013). Deliberate changes to gendered body motion influence basic social perceptions. Soc. Cogn. 31, 656–671. doi: 10.1521/soco.2013.31.6.656

[ref41] Longmore C. Tree J. (2013). Motion as a cue to face recognition: evidence from congenital prosopagnosia. Neuropsychologia 51, 864–875. doi: 10.1016/j.neuropsychologia.2013.01.022, PMID: 23391556

[ref42] Loula F. Prasad S. Harber K. Shiffrar M. (2005). Recognizing people from their movement. J. Exp. Psychol. Hum. Percept. Perform. 31, 210–220. doi: 10.1037/0096-1523.31.1.210, PMID: 15709874

[ref43] Mileva M. Burton A. M. (2018). Smiles in face matching: idiosyncratic information revealed through a smile improves unfamiliar face matching performance. Br. J. Psychol. 109, 799–811. doi: 10.1111/bjop.12318, PMID: 29920996

[ref44] Miller L. E. Saygin A. P. (2013). Individual differences in the perception of biological motion: links to social cognition and motor imagery. Cognition 128, 140–148. doi: 10.1016/j.cognition.2013.03.013, PMID: 23680791

[ref45] Munhall K. G. Buchan J. N. (2004). Something in the way she moves. Trends Cogn. Sci. 8, 51–53. doi: 10.1016/j.tics.2003.12.009, PMID: 15588806

[ref46] Noyes E. Hill M. Q. O’Toole A. J. (2018). Face recognition ability does not predict person identification performance: using individual data in the interpretation of group results. Cogn. Res. Princ. Implic. 3, 1–13. doi: 10.1186/s41235-018-0117-4 30009253 PMC6019422

[ref47] O’Toole A. J. Phillips P. J. Weimer S. Roark D. A. Ayyad J. Barwick R.. (2011). Recognizing people from dynamic and static faces and bodies: dissecting identity with a fusion approach. Vis. Res. 51, 74–83. doi: 10.1016/j.visres.2010.09.035 20969886

[ref48] O'Toole A. Roark D. (2011). “Memory for moving faces: the interplay of two recognition systems” in Dynamic faces: Insights from experiments and computation. eds. Curio C. Bülthoff H. H. Giese M. A. (MIT Press, Cambridge, Mass: Boston Review), 15–29.

[ref49] O'Toole A. J. Roark D. A. Abdi H. (2002). Recognizing moving faces: a psychological and neural synthesis. Trends Cogn. Sci. 6, 261–266. doi: 10.1016/S1364-6613(02)01908-3, PMID: 12039608

[ref50] Otsuka Y. Konishi Y. Kanazawa S. Yamaguchi M. K. Abdi H. O'Toole A. J. (2009). Recognition of moving and static faces by young infants. Child Dev. 80, 1259–1271. doi: 10.1111/j.1467-8624.2009.01330.x, PMID: 19630907

[ref51] Peterson L. Clifford C. Palmer C.. (2025). The role of (observed) gaze behaviour in identity recognition. Cognition 263:106222. doi: 10.1016/j.cognition.2025.106222 40580682

[ref52] Pike G. E. Kemp R. I. Towell N. A. Phillips K. C. (1997). Recognizing moving faces: the relative contribution of motion and perspective view information. Vis. Cogn. 4, 409–438. doi: 10.1080/713756769

[ref53] Pilz K. S. Thornton I. M. (2016). Idiosyncratic body motion influences person recognition. Vis. Cogn. 25, 539–549. doi: 10.1080/13506285.2016.1232327

[ref54] Pitcher D. Walsh V. Duchaine B. (2011). The role of the occipital face area in the cortical face perception network. Exp. Brain Res. 209, 481–493. doi: 10.1007/s00221-011-2579-1, PMID: 21318346

[ref55] Russell R. Duchaine B. Nakayama K. (2009). Super-recognizers: people with extraordinary face recognition ability. Psychon. Bull. Rev. 16, 252–257. doi: 10.3758/PBR.16.2.252, PMID: 19293090 PMC3904192

[ref56] Simhi N. Yovel G. (2017). The role of familiarization in dynamic person recognition. Vis. Cogn. 25, 550–562. doi: 10.1080/13506285.2017.1307298

[ref57] Simhi N. Yovel G. (2020). Can we recognize people based on their body-alone? The roles of body motion and whole person context. Vis. Res. 176, 91–99. doi: 10.1016/j.visres.2020.07.012, PMID: 32827880

[ref58] Smith H. M. Dunn A. K. Baguley T. Stacey P. C. (2016). Matching novel face and voice identity using static and dynamic facial images. Atten. Percept. Psychophys. 78, 868–879. doi: 10.3758/s13414-015-1045-8, PMID: 26732264 PMC4819615

[ref59] Steede L. L. Tree J. J. Hole G. J. (2007). I can’t recognize your face but I can recognize its movement. Cogn. Neuropsychol. 24, 451–466. doi: 10.1080/02643290701381879, PMID: 18416501

[ref60] Stevenage S. V. Nixon M. S. Vince K. (1999). Visual analysis of gait as a cue to identity. Appl. Cogn. Psychol. 13, 513–526. doi: 10.1002/(SICI)1099-0720(199912)13:6<513::AID-ACP616>3.0.CO;2-8

[ref61] Thornton I. M. Kourtzi Z. (2002). A matching advantage for dynamic human faces. Perception 31, 113–132. doi: 10.1068/p3300, PMID: 11922118

[ref62] Tickle-Degnen L. Zebrowitz L. A. Ma H. I. (2011). Culture, gender and health care stigma: practitioners' response to facial masking experienced by people with Parkinson's disease. Soc. Sci. Med. 73, 95–102. doi: 10.1016/j.socscimed.2011.05.008, PMID: 21664737 PMC3142938

[ref63] Troje N. F. (2002). Decomposing biological motion: a framework for analysis and synthesis of human gait patterns. J. Vis. 2:2. doi: 10.1167/2.5.2, PMID: 12678652

[ref64] Troje N. F. Westhoff C. Lavrov M. (2005). Person identification from biological motion: effects of structural and kinematic cues. Percept. Psychophys. 67, 667–675. doi: 10.3758/BF03193523, PMID: 16134460

[ref65] Valentine T. (1988). Upside-down faces: a review of the effect of inversion upon face recognition. Br. J. Psychol. 79, 471–491. doi: 10.1111/j.2044-8295.1988.tb02747.x, PMID: 3061544

[ref66] Valentine T. (1991). A unified account of the effects of distinctiveness, inversion, and race in face recognition. Q. J. Exp. Psychol. A. 43, 161–204. doi: 10.1080/14640749108400966, PMID: 1866456

[ref67] Vick S. J. Waller B. M. Parr L. A. Smith Pasqualini M. C. Bard K. A. (2007). A cross-species comparison of facial morphology and movement in humans and chimpanzees using the facial action coding system (FACS). J. Nonverbal Behav. 31, 1–20. doi: 10.1007/s10919-006-0017-z, PMID: 21188285 PMC3008553

[ref68] Wagner P. Malisz Z. Kopp S. (2014). Gesture and speech in interaction: an overview. Speech Comm. 57, 209–232. doi: 10.1016/j.specom.2013.09.008

[ref69] Whittle M. W. (2007). Gait analysis: An introduction. 4th Edn. Butterworth-Heinemann, USA: Butterworth-Heinemann.

[ref70] Wilmer J. B. (2017). Individual differences in face recognition: a decade of discovery. Curr. Dir. Psychol. Sci. 26, 225–230. doi: 10.1177/0963721417710693

[ref71] Xiao N. G. Perrotta S. Quinn P. C. Wang Z. Sun Y. H. P. Lee K. (2014). On the facilitative effects of face motion on face recognition and its development. Front. Psychol. 5:633. doi: 10.3389/fpsyg.2014.00633, PMID: 25009517 PMC4067594

[ref72] Xiao N. G. Quinn P. C. Ge L. Lee K. (2013). Elastic facial movement influences part-based but not holistic processing. J. Exp. Psychol. Hum. Percept. Perform. 39, 1457–1467. doi: 10.1037/a0031631, PMID: 23398253 PMC3889867

[ref73] Yovel G. O'Toole A. J. (2016). Recognizing people in motion. Trends Cogn. Sci. 20, 383–395. doi: 10.1016/j.tics.2016.02.005, PMID: 27016844

[ref74] Zhang Z. Hu M. Wang Y. (2011). “A survey of advances in biometric gait recognition” in Biometric recognition. CCBR 2011. eds. Sun Z. Lai J. Chen X. Tan T., Lecture Notes in Computer Science, vol. 7098 (Berlin, Heidelberg: Springer).

